# Factors associated with contraceptive use among women living with HIV in Canada: a controlled, cross-sectional study

**DOI:** 10.1186/s12978-021-01312-7

**Published:** 2022-01-05

**Authors:** Chadni C. Khondoker, Angela Kaida, Anna Marquez, Amber R. Campbell, Hélène C. F. Côté, Arianne Y. Albert, Neora Pick, Evelyn J. Maan, Emilie A. B. Russell, Jerilynn C. Prior, Chelsea Elwood, Jason Brophy, Melanie C. M. Murray, for the CIHR team grant on Cellular Aging, H. I. V. Comorbidities in Women, Children (CARMA), (CIHR HIV Clinical Trials Network study 277)

**Affiliations:** 1 Faculty of Medicine, University of British Columbia (UBC), Vancouver, BC Canada; 2 Oak Tree Clinic, BC Women’s Hospital, Vancouver, BC Canada; 3 Faculty of Health Sciences, Simon Fraser University, Burnaby, BC Canada; 4 Department of Experimental Medicine, UBC, Vancouver, BC Canada; 5 Women’s Health Research Institute, BC Women’s Hospital, E600B-4500 Oak Street, Vancouver, BC V6H 3N1 Canada; 6 Department of Pathology & Laboratory Medicine, UBC, Vancouver, BC Canada; 7 Centre for Menstrual Cycle and Ovulation Research, Endocrinology, Department of Medicine, UBC, Vancouver, BC Canada; 8 Department of Obstetrics and Gynecology, UBC, Vancouver, BC Canada; 9 Department of Pediatrics, Children’s Hospital of Eastern Ontario, University of Ottawa, Ottawa, ON Canada

**Keywords:** HIV, Contraception, Women, Canada

## Abstract

**Background:**

Multiple contraindications to combined hormonal contraceptives (CHC) use exist. The impact of these factors on contraceptive choice, particularly among women living with HIV (WLWH), is not well understood. We measured and compared the prevalence of contraceptive use and contraindications among WLWH and women not living with HIV (controls).

**Methods:**

We examined cross-sectional survey and medical chart data from 83 WLWH and 62 controls, aged 16–49 and sexually active, from 2013–2017. We compared the age-adjusted prevalence and types of contraceptives used in the last month and the proportion of women with CHC contraindications, including drug interactions, medical comorbidities, and smoking at ≥ 35 years old. All WLWH received care at an interdisciplinary, women-centred HIV clinic.

**Results:**

Compared to controls, WLWH were older (median [IQR)] 39 [34–43] vs 31 [23–41] years; p = 0.003), had less post-secondary education (37% vs 73%; p < 0.001), and more often had household income < $15,000/year (49% vs 30%; p = 0.006). WLWH trended to higher contraceptive prevalence than controls (80% vs 63%; p = 0.06 adjusted for age). Overall hormonal contraceptive use was similar. However, despite controlling for age, WLWH used CHC less (4% vs 18%; p = 0.006) than controls, and had more frequently undergone tubal ligation (12% vs 2%; p = 0.03). WLWH also experienced more CHC contraindications (54% vs 13%; p = 0.0001), including smoking at ≥ 35 years old (30% vs 6%; p = 0.0003) or a CHC-related drug interaction (all antiretroviral related) (25% vs 0%; p = 0.0001).

**Conclusions:**

WLWH attending our interdisciplinary clinic used hormonal contraception at similar rates as controls, though with different types. Differences may reflect different distributions of CHC contraindications. CHC contraindications present barriers to accessing the full range of contraceptive choices for WLWH. Guidelines and education for care providers and WLWH regarding contraceptive choices and drug interactions are needed, especially when care is provided without the benefit of an interdisciplinary women-centered healthcare team.

## Plain language summary

**Background:**

There are many reasons why individuals cannot use combined hormonal contraceptives (CHC). The impact of these reasons on contraceptive choice for women living with HIV (WLWH) are poorly understood. We measured and compared the prevalence of contraceptive choice and factors that may preclude their use in WLWH.

**Methods:**

We examined survey and medical chart data from 83 WLWH and 62 controls (women not living with HIV), aged 16–49 and sexually active, from 2013 to 2017. We compared the prevalence and types of contraceptives used in the last month and the proportion of women with factors that would not allow the use of CHC, including drug interactions, medical conditions, and smoking at ≥ 35 years old. All WLWH received care at a women-centred HIV clinic.

**Results:**

Compared to controls, WLWH were older, had less post-secondary education, and more often had household income < $15,000/year. WLWH were more likely to use contraception than controls. Overall hormonal contraceptive use was similar. However, even when accounting for age, WLWH used CHC less than controls, and had more frequently undergone tubal ligation. WLWH also had more reasons that would preclude the use of CHC contraindications including smoking at ≥ 35 years old or a CHC-related drug interaction.

**Conclusions:**

WLWH attending our interdisciplinary clinic used combined hormonal contraception at similar rates as controls, though with different types. Differences may reflect the fact that WLWH more often have factors that do not allow the safe use of CHC. Guidelines and education for care providers and WLWH regarding contraceptive choices and drug interactions are needed.

## Background

Longer life expectancy and improved health status due to availability of combination antiretroviral therapy [1] has contributed to an increased rate of pregnancy among women living with HIV (WLWH) [2]. A retrospective analysis involving 1165 Canadian WLWH reported that 61% of pregnancies were unintended [2]; a value that exceeds the Canadian average of up to 40% [3]. Unintended pregnancies are associated with negative outcomes such as delayed antenatal care and low birth weight [4]. These risks are elevated for WLWH as there is potential for vertical HIV transmission [5]. Whilst the primary goal of contraception is often pregnancy prevention [6], contraceptive choice is influenced by life circumstances, patient-centered goals and factors such as cost, religious beliefs, side effects, and protection from sexually transmitted infections [6, 7]. Concurrently, healthcare provider (HCP) consideration of patient comorbidities, drug interactions, and behavioral factors also influence contraceptive choice [8]. Access to a full range of contraceptive options supports a woman’s reproductive rights [9]. Furthermore, preventing unintended pregnancies decreases maternal and infant morbidity and mortality risks [4] and the probability of vertical HIV transmission [5]. Thus, safe contraceptive options and choice are imperative for WLWH.

The World Health Organization (WHO) guidelines state that WLWH should be offered a full range of contraceptive options [10]; however previous studies assessing contraceptive choice in WLWH have shown that the range of methods used in Canada is limited, particularly related to hormone-based contraceptives use [11, 12]. Approximately 9–21% of sexually active WLWH in Canada use hormonal contraceptives (injectable depo-medroxyprogesterone, combined hormonal contraceptives [CHC] by vaginal ring, patch or oral delivery [7], or levonorgestrel-releasing intrauterine device [LNG-IUD]) [11, 12], a rate less than half of that used by the general Canadian population (44%) [13]. Specific antiretroviral medications (ARVs), other drugs, certain medical comorbidities, and smoking when ≥ 35 years of age are contraindications to using CHC [8, 10, 14, 15]. Assessing patient choice and associated medical factors is an important step toward understanding prescribing practices and contraceptive methods used among WLWH.

Several commonly used ARVs reduce the efficacy of CHC [8, 10, 14, 15]. These ARVs may induce the liver CYP450 3A4 system that metabolizes estrogen, thus accelerating its clearance [7]. Use of efavirenz, darunavir, or combined use of lopinavir/ritonavir in tandem with CHC is contraindicated, while elvitegravir or atazanavir require a higher dose ethinyl estradiol-containing CHC to be effective [16–20]. ARV treatment guidelines advise prescribers to either avoid simultaneous CHC use, or to provide a higher dose ethinyl estradiol-containing CHC when also taking interacting ARVs [15]. Other contraindications include: anticonvulsants, rifamycins (such as rifampin), and smoking in women ≥ 35 years old [8, 10]. World Health Organization (WHO) guidelines state that use of CHC is contraindicated in the presence of any of the following comorbidities: hypertension, deep vein thrombosis/pulmonary embolism, diabetes with retinopathy, neuropathy, or nephropathy, diabetes duration > 20 years, migraine with aura, liver tumour, myocardial infarction, stroke, severe cirrhosis/liver failure, active cancer or history of breast cancer [8]. If the prevalence of these CHC-related contraindications is greater among WLWH, this may influence contraceptive prescribing practices, and therefore partially explain the low rate of hormonal contraceptive use found in previous studies.

Previous cohort studies assessing factors associated with contraceptive use in Canada looked at psychological, socio-behavioral, demographic, sexual, and reproductive characteristics [11, 12]. However, little is known about contraceptive choice in relation to medical comorbidities, drug contraindications, and smoking. We assessed and compared the prevalence of contraceptive choice among WLWH and controls (women not living with HIV) in the Children and Women: Antiretrovirals and Markers of Aging (CARMA) cohort. We then examined the relationship of medical comorbidities, drug contraindications, and smoking with participant self-reported contraceptive use. For the purpose of this paper, whenever we use the term “women”, we are referring to “cis-women”.

## Methods

### Study design and setting

The CARMA study is an ongoing prospective cohort study of WLWH and HIV-negative controls that aims to investigate the effects of HIV and ARV therapy on cellular aging in women and children. The CARMA-ENDO study is a cross-sectional sub-study of CARMA aimed at examining the endocrine, metabolic and reproductive health of women and female youth living with HIV (LWH). The CARMA-ENDO study enrolled non-pregnant women and girls (age ≥ 12 years) both LWH and HIV-negative between January 2013 and August 2017. All individuals who had participated previously in the CARMA cohort who had consented to being contacted for future studies were invited to participate in the endocrine sub-study during regularly scheduled clinic appointments (if HIV-positive) or were contacted by email or phone if HIV-negative. New WLWH participants were recruited during routine HIV care visits. New controls were recruited through advertisements strategically placed to recruit participants with sociodemographic characteristics similar to participants LWH. All visits occurred at, an interdisciplinary HIV center in Vancouver, BC, which provides specialized HIV care to women, children, and their families [21].

### Inclusion/exclusion criteria

**Fig. 1:**
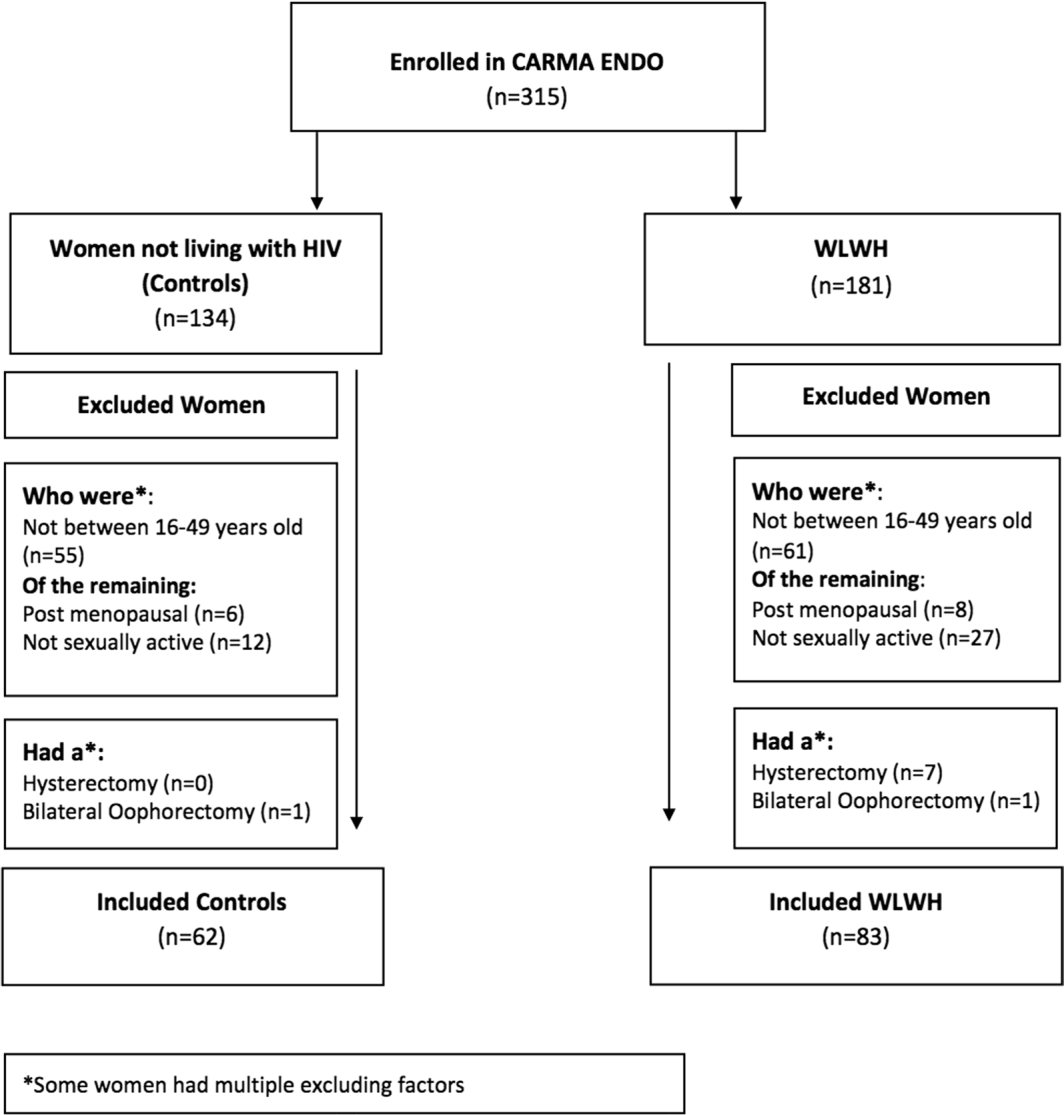
Consort diagram of CARMA-ENDO cohort Contraceptive Choice Study

For this cross-sectional analysis, we included all WLWH and controls aged 16–49 years old at the time of the CARMA-ENDO visit who were not menopausal, had not had a hysterectomy or bilateral oophorectomy, and were sexually active in the past 6 months (hence could become pregnant). See (Fig. 1) for study participant selection. As this is an explorotory post-hoc analysis of the larger CARMA cohort study, our sample size is comprised of those participants that met criteria for inclusion. The study was approved by the University of British Columbia Children & Women’s Research Ethics Board (H08-02018).

### Data collection procedures

Relevant demographic, clinical, laboratory, and substance exposure data were collected. Participants completed structured questionnaires administered by research assistants, and blood samples were collected on site. Study data were collected and managed using REDCap electronic data capture tools hosted at B.C Children’s Health Research Institute [22].

### Measures

Participants reported all contraceptive methods used in the past month, and these were further categorized into various types of hormonal methods: levonorgestrel-releasing intrauterine device (LNG-IUD), progestin only (oral form, birth control implant and injectables) and CHC (as a patch, oral form or vaginal ring). Type of oral contraceptive used (progestin only or combined) was self reported or identified through chart review. The Society of Obstetrics and Gynecology Canada categorizes contraceptives into a 3-tier model based on effectiveness [23]; top tier methods (IUD, birth control implant, partner vasectomy, and tubal ligation) are recommended to most effectively prevent pregnancy [23]. In keeping with this format, contraceptive methods were also grouped as being “top-tier” or long acting reversible contraceptives (LARC). LARC methods include IUD and birth control implants. ARV use was determined via chart review for all WLWH. Current use of tobacco, and prescription drugs was determined via self-report. History of medical contraindications as per WHO guidance were determined through self-report (medical history, medication profile). For WLWH, medical history was validated with, but not based on, chart review; self-report was used for controls. Medical comorbidity diagnoses were made if participants self reported having been previously diagnosed by a physician or if they took medication consistent with the diagnosis. Menopause was defined as self-report of amenorrhea for ≥ 1 year coupled with a measured follicle stimulating hormone (FSH) of > 25 milli-International Units/mL as per criteria previously published [24]. This method of defining menopause was chosen as a significant portion of WLWH in the CARMA cohort are amenorrhoeic in the absence of menopause [25]. For women reporting ≥ 12 months of amenorrhea, FSH was measured from study visit samples through either the hospital lab or using the Enzo Life Sciences FSH ELISA kit (ab108641 Farmingdale, New York) [26]. A coefficient was applied to ensure FSH data from both sources were correlated (slope = 0.99, r^2^ = 0.8). HIV viral load and CD4 were collected.

### Statistical analysis

Clinical and demographic characteristics were summarized using descriptive statistics, frequencies and percentages for categorical variables, means with standard deviation, or medians with interquartile ranges for continuous variables as appropriate. Clinical and demographic variables were compared between WLWH and controls using t-tests for continuous variables, and Fisher’s exact tests for categorical variables. We used logistic regressions to compare the prevalence of specific contraceptive types (where the combined number of users was at least 10) between the groups, controlling for age as a covariate, and additionally including any contraindication to CHC (smoking at ≥ 35 years of age old, medical or drug contraindication to CHC use, along with age as a covariate). Due to limited data, when the combined number of participants that used a contraceptive method was less than 10, descriptive reporting was alternatively used. All statistical tests were two-sided and were considered statistically significant at p < 0.05.

## Results

### Demographics

We analyzed data from 145 premenopausal sexually active women that included 83 WLWH and 62 controls between 16–49 years of age. These numbers represent those who met our inclusion criteria, or 46% of the full cohort. Compared with controls, WLWH were older, had less education, and were more likely to have an income < $15,000/year (Table 1). Among WLWH, 92% were on ARVs, and mean (± SD) CD4 count was 541 (± 337) cells/mm^3^. Of those women on ARVs, 75% had an undetectable HIV viral load (< 40 copies/mL) at study visit.

**Table 1:** Demographics and clinical characteristics of women enrolled in the CARMA Cohort

	Total	Controls	WLWH	P-value
	n = 145	n = 62	n = 83	
Age, median [IQR]	37 [28–43]	31 [23–41]	39 [34–43]	**0.003**
Race/Ethnicity, n (%)				
White	59 (45)	28 (45)	31 (37)	**0.02**
Indigenous	39 (37)	11 (18)	28 (34)	
African/Caribbean/Black	24 (17)	8 (13)	16 (19)	
Other/unknown	23 (17)	15 (24)	8 (10)	
Education, n (%)				
≤ High School Graduate	59 (41)	16 (26)	43 (52)	** < 0.001**
Some Post Secondary	76 (52)	45 (73)	31 (37)	
Income, n (%)				
< $15,000/y	60 (42)	19 (30)	41 (49)	**0.006**
≥ $15,000/y	75 (52)	42 (68)	33 (37)	
Number of Pregnancies, median [IQR]	2 [0–4]	0 [0–2]	3 [1–5]	** < 0.001**
Current Smoker, n (%)	43 (30)	9 (15)	34 (41)	** < 0.001**
Illicit Drug Use^◊^, n (%)	8 (6)	1 (2)	7 (8)	0.1
Prescribed Opiate Use, n (%)	31(21)	2 (3)	29 (35)	** < 0.001**

Bolded characters represent statistically significant values

Women Living with HIV (WLWH); IQR (Interquartile range); ^◊^Illicit Drug Use: Current use of cocaine, crack, crystal meth, heroin, unprescribed opiates

### Contraindications

With respect to contraindications to CHC use, WLWH were more likely to be current tobacco smokers ≥ 35 years of age and to have a drug contraindication to CHC (Table 2). The presence of medical contraindications between WLWH and controls were similar (Table 2). All noted drug contraindications for WLWH resulted from ARV medication use: efavirenz (10), darunavir (9) and lopinavir/ritonavir (2). Overall, 54% of WLWH had at least one contraindication to CHC use compared to 13% of controls (p = 0.0001) (Table 2).

**Table 2:** Prevalence of Contraindications to Combined Hormonal Contraceptives

Type of Contraindication	Controls (n = 62)	WLWH (n = 83)	p-value
Medical Contraindication^@^, n (%)	6 (10%)	14 (17%)	0.14
Current Tobacco Smoker ≥ 35y, n (%)	4(6%)	25 (30%)	**0.0008**
Drug Contraindication*, n (%)	0 (0%)	21 (25%)	**0.0001**
≥ 1 Contraindication to CHC, n (%)	8 (13%)	45 (54%)	**0.0001**

Bolded characters represent statistically significant values

Women living with HIV (WLWH), Combined Hormonal Contraceptive (CHC)

^@^Medical Contraindication: Hypertension, deep vein thrombosis/pulmonary embolism, diabetes with retinopathy, neuropathy, or nephropathy, diabetes > 20 years, migraine with aura, liver tumour, heart attack/myocardial infarction, stroke, severe cirrhosis, active cancer or any history of breast cancer

*Drug Contraindication: Current use of rifampicin, rifabutin, phenytoin, carbamazepine, topiramate, phenobarbital, oxcarbazepine, primidone, lamotrigine, efavirenz, darunavir, or combined use of ritonavir/lopinavir

### Contraception

**Fig. 2:**
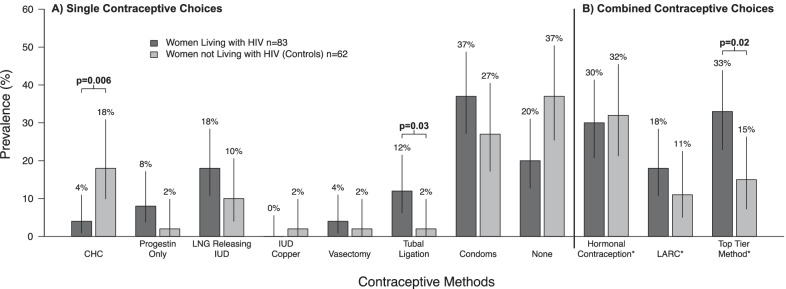
Prevalence of Contraceptive Method Used.** A** Single Contraceptive Choices: Combined Hormonal contraceptive (CHC); Progestin only: injectables, implants; Levonorgestrel (LNG) intrauterine device (IUD); IUD Copper; Vasectomy; Tubal Ligation; Condoms; None (no use of any method including alternative methods e.g. withdrawal). **B** Combined Contraceptive Choices: Hormonal Contraception: Combined hormonal contraceptive (CHC), progestin only, and levonorgestrel intrauterine device (IUD); Long acting reversible contraceptives (LARC):IUD, and birth control implant; Top Tier Method: vasectomy, tubal ligation, birth control implant, intrauterine device. *Composite Method, **Only significant p values are shown and are adjusted for age)

Overall prevalence of contraceptive use was similar between WLWH and controls after adjusting for age (80% vs. 63%; p = 0.06). Condoms were the most commonly used form of contraception among both groups, followed by the LNG-IUD, and tubal ligation in WLWH and by CHC and LNG-IUD in controls (Fig. 2). Overall use of hormonal contraceptives was similar between WLWH and controls (adjusted p = 0.94) however the groups differed in types of hormonal contraceptive methods used. After adjusting for age, WLWH were more likely to employ a top-tier method (AOR 2.78 (95%CI 1.20–6.67); p = 0.02), or to have had a tubal ligation (AOR 6.25 (95%CI 1.14–100); p = 0.03) than controls (Table 3). However, with adjustment for age, WLWH were 82% less likely to use CHC (AOR 0.18 (95%CI 0.04–0.63); p-0.006) compared with controls. When we also adjusted for the presence of ≥ 1 contraindication to CHC, the odds ratios for CHC use was augmented and became non-significant; (AOR 0.28 (95%CI (0.06–1.05)); p = 0.064) (Table 3), the AOR for tubal ligation decreased but remained significant, and the AOR for use of top-tier methods became non-significant (AOR 2.17 (95% CI 0.87–5.56); p = 0.10). As data for type of oral contraceptive used for 2/12 HIV-negative women were not available, they were not included in the analysis of CHC use.

**Table 3:** Crude and Adjusted Odds Ratios (AOR) for Contraceptive Methods used by WLWH and HIV-negative controls

Method	n/N	Crude OR (95%CI)	Crude p-value	Age AOR (95% CI)	Age adjusted p-value	AOR* (95%CI)	Adjusted* p-value
Combined Hormonal Contraceptives HIV-negative (ref) WLWH	11/60 3/83	0.17 (0.03–0.68)	0.004	0.18 (0.04–0.63)	0.006	0.28 (0.06–1.05)	0.06
Tubal Ligation HIV-negative (ref) WLWH	1/62 10/83	8.33 (1.54–100)	**0.02**	6.25 (1.14–100)	**0.03**	7.14 (1.14–100)	**0.03**
Top Tier method HIV-negative (ref) WLWH	9/62 27/83	3.03 (1.33–7.14)	**0.02**	2.78 (1.20–6.67)	**0.02**	2.17 (0.87–5.56)	0.10

Bolded characters represent statistically significant values

*Adjusted for age and presence of ≥ 1 contraindication to CHC. Top tier method (vasectomy, tubal ligation, birth control implant, or intrauterine device); OR (odds ratio); AOR (adjusted odds ratio); WLWH (women living with HIV)

Data regarding timing of tubal ligation was available and assessed in 8/10 WLWH who experienced it; all occurred after women had borne at least 2 children. The timing of tubal ligations with respect to HIV diagnosis date was available for 5/10 of the woman. Tubal ligations occurred ≥ 4 years after diagnosis for 4/5 women, while 1/5 woman underwent the procedure during the first year after HIV diagnosis.

## Discussion

We found WLWH and controls used contraception at similar rates. Use of any hormone-based contraceptive (including CHC, progestin-only pills, progestin implants and LNG-IUD) among WLWH and controls in our cohort was similar. However, the type of hormonal contraceptive methods used differed between WLWH and controls, with WLWH much less likely to use CHC. Previous Canadian studies have showed differences in use of hormonal methods between the WLWH studied and the general population [11, 12]. By only assessing overall hormonal contraceptive use, those studies may have masked differences in the types of hormonal methods used. Use of hormonal contraception by both WLWH and controls was lower than the Canadian average of 44% [13]. This is consistent with a recent analysis of contraceptive use in WLWH and HIV-negative women in the United States, which has shown similar use of hormonal contraceptives between the two groups [27]. Reasons for this change is unclear, however may be related to revision of guidance statements by the World Health Organization, which now state that WLWH can use the full range of contraceptive options, including hormonal contraceptives, with no increased risk of HIV acquisition [28]. Previous to this, the WHO recommended extra consideration for use of progestin only injectable contraceptives, which may have discouraged the use of hormonal contraceptives in WLWH [28].

In our cohort, after controlling for age differences between the groups, WLWH were 82% less likely (AOR 0.18) to use CHC versus controls. However, when controlling for contraindications, the AOR rose to 0.28 and becomes non-significant, suggesting this factor accounts for at least part of the lower prevalence of CHC use among WLWH.

With the exception of CHCs, WLWH in our cohort are using a wider range of methods than controls, and also compared to WLWH described in previous studies [11, 12]. Most notably, use of LNG-IUD in WLWH was greater here than documented previously, and may relate to the interdisciplinary care provided to participants that involves obstetrics/gynecologic care and internal funding for LNG-IUD [21]. Interdisciplinary healthcare models, have been shown to improve health outcomes for people LWH [29]. In places where an interdisciplinary women-centered healthcare model is not possible, clear guidelines emphasizing drug interactions with contraception along with alternative contraceptive methods and dosing options need to be made available for HCP to ensure consistency. Currently, primary care guidelines are available to support care and treatment programs for people LWH in British Columbia [30]. However, although these guidelines briefly discuss contraceptive choice and potential drug interactions, HCP are left with little direction on how to appropriately amend prescribing practices to best support their patients [30]. Furthermore, in primary care settings, discussions regarding ARV regimens often take precedence. However, when caring for WLWH between 16 and 49 years of age, comprehensive care includes addressing contraception options in tandem with HIV-specific care [30].

Interestingly, after controlling for age, use of top-tier contraceptive methods was greater among WLWH compared with controls, suggesting that the age difference between the groups was not the only factor involved in the difference between these groups. Among WLWH, a high prevalence of tubal ligations and LNG-IUD use may help to explain this difference. When we also controlled for the presence of a contraindication to CHC, WLWH and controls had similar use of top-tier contraceptive methods. This may reflect the fact that, when CHC is not appropriate due to contraindications, these LARC methods are a higher portion of the options left to the prescriber.

Tubal ligations were more prevalent among WLWH than controls. History of tubal ligation was largely independent of age or CHC contraindications. Reasons for higher rate of tubal ligation in women living with HIV were unclear, however previous studies have found that both age and parity influence contraceptive use [13, 30]. In our study WLWH were older and had higher parity. As women mature and have children, they may opt for longer acting or irreversible contraceptive methods to better align with their family planning goals [13, 31].

Furthermore, WLWH were more likely to be smokers over the age of 35 years old. This is consistent with previous studies done in the United States that found the rate of smoking to be up to 3 times higher in people living with HIV than the general population [32].^.^ We included this variable in our analysis as smoking while over the age of 35 years old is a contraindication to the use of CHC. Smoking and use of CHC has a synergistic effect on the risk of negative hematologic outcomes [33, 34] which greatly reduces the applicability of CHC use in WLWH. Therefore, the increased rate of smoking in WLWH may partially contribute to lower use of CHC in this group. The difference in proportion of women who were smokers over the age of 35 in each group may relate to the fact that WLWH in the study were older. As we recognized that age may be a confounding factor, we controlled for age in our analysis. We found that despite controlling for age, CHC use was still less among WLWH compared to control women (Table 3).

## Limitations

Contraceptive method use was determined via self-report, but the reasons for specific contraceptive choices were not collected and remain unknown. For this reason and the cross-sectional nature of this study, we can only examine correlations and not causation. Additionally, the health care for WLWH participants in this study was at an interdisciplinary women-centred HIV clinic in a tertiary healthcare centre, therefore contraceptive counselling and decision-making may not reflect practices at other care settings. Furthermore, the power of our study was limited by our small sample size, and there are potential relationships that we could not detect as a result of this. Despite our best efforts, we were unable to identify the type of oral contraceptive pill used for 2/12 HIV-negative participants, thus reducing the sample size for our analysis of CHC use. Finally, WLWH in our cohort were older and had higher parity than controls; both of these factors have previously been found to be associated with contraceptive choice and may influence the method used [13, 31].

## Strengths

By assessing the prevalence of contraindications to CHC use, our study provides insight on the rates and potential reasons for contraceptive choices and may partially explain differences seen in contraceptive methods used in previous studies.

## Conclusion

Hormonal contraceptive use in our cohort was similar between WLWH and controls. The range of contraceptive methods used by WLWH was wider than in controls and than seen in previous studies. This may relate to the fact that WLWH in this study received care at an interdisciplinary healthcare clinic which can help facilitate decisions regarding contraceptive choice. Guidelines and education for HCP and WLWH themselves regarding contraceptive choices and drug interactions are needed, especially when care is provided without the benefit of an interdisciplinary women-centered care team. Guidelines would offer HCP an essential tool to support the contraceptive choices and reproductive health rights of WLWH.

## Data Availability

The datasets generated and/or analysed during the current study are not publicly available due to the fact participants were not consented to have this data be publicly available, but the datasets are available from the corresponding author on reasonable request.

## Abbreviations

CHC: Combined hormonal contraceptives
WLWH: Women living with HIV
HCP: Healthcare provider
WHO: World Health Organization
LNG-IUD: Levonorgestrel-releasing intrauterine device
ARVs: Antiretrovirals
CARMA: Children and Women: Antiretrovirals and Markers of Aging
AOR: Adjusted Odds Ratio
LARC: Long acting reversible contraceptives
FSH: Follicle stimulating hormone
